# Biology and Therapeutic Targets of Colorectal Serrated Adenocarcinoma; Clues for a Histologically Based Treatment against an Aggressive Tumor

**DOI:** 10.3390/ijms21061991

**Published:** 2020-03-14

**Authors:** Begoña Alburquerque-González, Fernando F. López-Calderón, María Dolores López-Abellán, Ángel Esteban-Gil, José García-Solano, Pablo Conesa-Zamora

**Affiliations:** 1 Department of Histology and Pathology, Faculty of Life Sciences, Universidad Católica de Murcia (UCAM), 30107 Murcia, Spain; begoalbur@gmail.com (B.A.-G.); davolef@gmail.com (F.F.L.-C.); jgarcia652@ucam.edu (J.G.-S.); 2 Department of Pathology, Santa Lucía General University Hospital (HGUSL), Calle Mezquita sn, 30202 Cartagena, Spain; 3 Department of Clinical Analysis, Santa Lucía General University Hospital (HGUSL), Calle Mezquita sn, 30202 Cartagena, Spain; mariadolores.lopezabellan@outlook.es; 4 Biomedical Informatics & Bioinformatics Platform, Institute for Biomedical Research of Murcia (IMIB)/Foundation for Healthcare Training & Research of the Region of Murcia (FFIS), 30003 Murcia, Spain; angel.esteban@ffis.es; 5 Research Group on Molecular Pathology and Pharmacogenetics, Institute for Biomedical Research of Murcia (IMIB), Calle Mezquita sn, 30202 Cartagena, Spain

**Keywords:** colorectal cancer, serrated adenocarcinoma, angiogenesis, immune response, invasive front, molecular targets

## Abstract

Serrated adenocarcinoma (SAC) is a tumor recognized by the WHO as a histological subtype accounting for around 9% of colorectal carcinomas. Compared to conventional carcinomas, SACs are characterized by a worse prognosis, weak development of the immune response, an active invasive front and a frequent resistance to targeted therapy due to a high occurrence of KRAS or BRAF mutation. Nonetheless, several high-throughput studies have recently been carried out unveiling the biology of this cancer and identifying potential molecular targets, favoring a future histologically based treatment. This review revises the current evidence, aiming to propose potential molecular targets and specific treatments for this aggressive tumor.

## 1. Introduction

**Figure 1:**
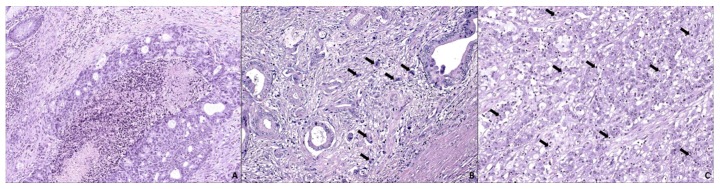
Differential histological features of histological subtypes of colorectal carcinoma. (**A**) Conventional carcinoma (CC) showing a cribriform gland pattern with basophilic cytoplasm, non-stratified nuclei, lobular dirty necrosis (centre) and lymphocytic infiltrates (upper-left corner). (**B**) Serrated adenocarcinoma (SAC) with typical vesicular stratified nuclei, eosinophilic cytoplasm and serrated lumen (left gland) with weak lymphocytic infiltration, tumor budding (black arrows) and desmoplastic stroma. (**C**) Colorectal carcinoma (CRC) with histological and molecular features of microsatellite instability (hmMSI-H) characterized by a medullar “solid” pattern and abundant intraepithelial tumor-infiltrating lymphocytes (black arrows) 20× original magnification. (Source: Authors).

Colorectal carcinoma (CRC) accounts for the third–most frequent cause of cancer death worldwide [1]. This tumor develops from precursor lesions (polyps/adenomas) following at least two pathological routes; the most common one, the adenoma–carcinoma sequence, is typically characterized by chromosomal instability and microsatellite stability leading to the development of conventional carcinoma (CC). Less is known about the serrated pathway, in which a high level of microsatellite instability (MSI-H), high frequency of BRAF mutation and the CpG island methylation phenotype (CIMP) seem to be the leading carcinogenic causes, although significant proportions of SAC are KRAS-mutated and microsatellite-stable [2,3]. These alterations contribute to the development of serrated adenocarcinoma (SAC) and the CRC showing histological and molecular features of MSI-H (hmMSI-H) [4], which are considered as endpoints of this pathway [5,6]. MSI-H is a manifestation of a deficiency in DNA mismatch repair mechanism which has been associated with histological features including the presence of signet-ring cells, mucine, tumor heterogeneity with a medullary component, poor differentiation, “pushing” type tumor growth pattern, peri- and intra-tumoral infiltrating lymphocytes, and “Crohn-like” inflammatory response [7]. SAC has been recognized in the latest WHO classifications of digestive tumors [8,9], accounts for 7.5%–9.1% of all CRCs and is diagnosed based on histological criteria put forward by Mäkinen [10,11], the serrated appearance of the epithelial glandular crypts being the most characteristic feature (Figure 1). This morphological pattern seems to be due to apoptotic evasion that causes the transformed epithelium to proliferate laterally, adopting a sawtooth growth pattern [8,10]. In fact, apoptosis-related genes were found to be enriched when comparing the expression signature of SAC with that of CC, and the immunohistochemical expression of hippocalcin, an anti-apoptotic protein, was proposed as a biomarker of SAC [12].

SAC develops from serrated adenomas through a fast process. Therefore, it is not unexpected that SACs are frequently found as “interval” CRCs and are related with synchronous and metachronous advanced colorectal tumors [13]. In addition to this higher abundance of synchronous carcinoma compared to CC, distant polyps present in surgical resections containing SAC frequently show serrated morphology, thus suggesting a bystander effect or individual predisposition for serrated carcinogenesis [11]. At this point it, is noteworthy that smoking and alcohol abuse have been associated with the development of the serrated carcinogenesis pathway [14,15,16], as well as the presence of *Fusobacterium nucleatum* in the patient’s feces [17].

Not surprisingly, SAC has a worse prognosis than CC [11], and in-depth studies have revealed that, compared to CC, SAC shows a more prominent invasive front, which is characterized by abundant histologically adverse prognostic factors such as tumor budding, cytoplasmic pseudofragments and an invasive tumor infiltrating pattern [18]. Consequently, the loss of E-cadherin expression and the increase in mesenchymal markers are more evident in SAC than in CC [19]. These histological and immunohistochemical manifestations of the invasive activity of SAC tumor cells were further confirmed by analyzing the molecular signatures of SAC compared to CC, where functions associated with cytoskeleton rearrangement and small GTPases’ activity were frequently enriched in SAC [12,20]. Intriguingly, the epithelial mesenchymal transition (EMT) in SAC does not seem to involve the canonical Wnt/β-catenin, as the nuclear expression of β-catenin was lower in SAC than in CC. In fact, the same β-catenin nuclear exclusion was observed by Davies et al. in serrated adenomas spontaneously developed in transgenic mice (*Pten^fl/fl^Kras^LSL/+^*) [21]. Other mouse serrated models that abolish atypical protein kinase C (aPKC) (*Prkci^fl/fl^ Prkcz^fl/fl^Villin-cre*) drive serrated intestinal cancer, also showing a lack of nuclear staining of β-catenin [22].

This histologically active invasive front and molecularly manifest epithelial-mesenchymal transition have led SAC to be considered as belonging to the so-called “mesenchymal” or type 4 comprehensive consensus molecular subtype (CMS4) of CRC [23], which is characterized by microsatellite stability, weak immune response and worse prognosis. Histological and molecular classifications of CRC do not perfectly overlap, since another subtype of SAC, termed “classical serrated CRC”, has been proposed that belongs to the CMS1 subtype and is characterized by BRAF mutation, CIMP, microsatellite instability (MSI), higher immune response and better prognosis than the “mesenchymal” SAC type [23].

**Figure 2:**
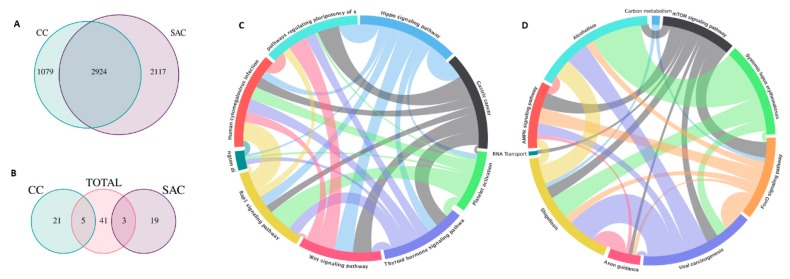
Differentially enriched functions in the SAC and CC transcriptomes. (**A**) Venn diagram displaying the number of common genes up- or down-regulated in SAC and CC compared to normal adjacent mucosa. Differential expression analysis has been performed on normalized data with the Linear Models for Microarray Data (Limma) package by Bioconductor [29]. Using Limma, two comparisons were made between 5 normal and 12 CC samples and the same 5 normal and 13 SAC samples. (**B**) The Kegga method from Limma was used to perform this analysis, which obtained 49 pathways significantly enriched for shared differentially expressed genes in both groups: 26 pathways for CC (5 of them shared) and 22 pathways for SAC (3 of them shared). As expected, there were no shared pathways between the different genes of the groups CC and SAC. For the false discovery rate (FDR), the Benjamini and Hochberg method was used to get corrected *p*-values [30]. Differentially enriched functions in CC (**C**), including Wnt-signalling and in SAC (**D**), including those lifestyle-, neural-, immune-hypoxia-related (**D**) are shown. The VEGF signalling pathway was close to significance in SAC, with 7 differentially expressed genes (MAPKAPK3, VEGFA, PIK3R2, RAF1, PLA2G4C, PPP3CB and PRKCB) and some related hypoxia-associated pathways (AMPK and mTOR signalling). FSCN1 is differentially expressed in the comparison Normal vs. SAC, but it is not in Normal vs. CC.

High-throughput technologies have yielded interesting findings about SAC biology and potential diagnostic markers for SAC. Laiho et al. performed functional enrichment analysis to identify categories with significant enrichment based on the genes differentially expressed in tumors with serrated and non-serrated morphology. Five out of nine categories were linked to morphogenesis, organogenesis and membrane-associated genes. Two genes (*EPHB2*, *PTCH*) involved in cell migration and morphogenesis were found to be downregulated, whereas one gene (*HIF1A*) related to angiogenesis was upregulated in SAC compared to CC [24]. In this line, angiogenesis and signalling of VEGF (an effector gene of HIF-1α transcriptional activity) have been found by other authors to be characteristic enriched functions associated with SAC’s molecular signature [12,20]. Consequently, the immunohistochemical expression of HIF-1α and the presence of microvascular density were significantly more abundant in SAC than in CC [25]. It is noteworthy that other enriched functions associated with SAC in molecular profiling studies are labelled as neural-related and, more remarkably, as related to immune response against either autoantigens or pathogens [12,20]. Freely accessible data from our research group have been retrieved from the ColPortal repository [26] and reanalyzed for function enrichment compared to CC (Figure 2). Common enriched functions found in SAC and CC compared to normal adjacent mucosa are presented as Figure S1. The lack of immune response is another important feature of SAC that has a histological manifestation when looking at this tumor invasive front where, compared to CC, there is a significantly lower presence of peritumoral and intratumoral lymphocytic infiltrates [18]. This poorer immune response of SAC is even more dramatic when compared to hmMSI-H, and therefore, transcriptome, micro-transcriptome and methylome studies have supported this histological observation [6,27,28]. These facts, added to the fact that most SACs are microsatellite-stable [2,3], do not make SAC a good candidate for biological therapy targeting the immune-checkpoint.

Independently studies on Finnish and Spanish CRC patients have shown that a high combined mutation rate of KRAS and BRAF in SAC (78.6% for the Finnish and 68.5% for the Spanish cohorts) indicates that mitogen-activated protein kinase (MAPK) activation plays a key role in the serrated pathway. These percentages in SAC are higher than those found in matched CC (40% in the Finnish and 33.1% in the Spanish patients), thus implying that most SACs will not benefit from anti-EGFR treatment, the major recent breakthrough in targeted therapy for metastatic CRC (mCRC) [2,3].

Given the aggressive behavior of SAC, its poorer outcome and the higher resistance to anti-EGFR compared to CC, there is an urgent necessity to develop targeted therapies for SAC. This review summarizes the current evidence about potential molecular targets that could be a basis for a treatment tailored to this histological subtype of CRC. Main potential therapeutic approaches against SAC histology or SAC-associated biomarkers are summarized in Table 1.

## 2. SAC Shows an Upregulation of Angiogenesis Markers

Clinically, antiangiogenic (anti-VEGF) therapy for mCRC has become the standard therapy in combination with several cytotoxic drugs [43]. Given the significant role of angiogenesis in SAC, as demonstrated by the overexpression of Hypoxia-inducible Factor 1α (HIF-1α) and VEGF and high microvessel density in serrated adenocarcinoma compared to conventional colorectal adenocarcinoma, these molecular biomarkers could be the key to new anti-angiogenic therapies specific to SAC.

Due to the characteristics of the tumor vascular network, tumor vessels exhibit immaturity and excessive permeability, leading to poor perfusion and increased hypoxia in the tumor microenvironment [44]. Under hypoxic conditions, HIF-1 is able to escape degradation and increases the transcription of genes involved angiogenesis, survival, cell proliferation, cell migration and glucose metabolism [45,46]. The angiogenic growth factors induced by inadequate local perfusion and chronic hypoxia in tumor tissue can also result in reduced leukocyte recruitment and resistance to both chemotherapy and radiotherapy [47]. This lack of immune activation is also characteristic of SAC and is discussed below.

HIF-1 expression can also be upregulated by oxygen-independent mechanisms triggered by oncogenes (e.g., EGFR, RAS and BRAF) or growth factors that stimulate MAPK, mTOR and PI-3K/Akt pathways, and by a lack of tumor suppressors such as VHL and PTEN [48,49].

Angiogenesis in CRC is induced by the HIF-1α subunit through the activation of expression of the HIF-1 target gene vascular endothelial growth factor (VEGF), as shown in a number of studies using immunohistochemistry in CRC tissue specimens [50].

Previous results on HIF-1α expression in normal colonic mucosa have shown its presence in cells in the upper part of the crypts and cells of the proliferative component in normal colorectal mucosa [51]. HIF-1α mRNA and/or protein is detected in both adenomas and CRCs, being more commonly expressed in adenocarcinomas than in adenomas, as a number of immunohistochemical studies indicate [52,53]. Thus, HIF-1α expression is also frequently correlated with the disease stage [50]. Rigopoulos et al. have also demonstrated significant association between VEGF and HIF-1α immunohistochemical expression, as well as deregulation of the EGFR/VEGF/HIF-1α signalling pathway in colon adenocarcinoma [54].

Importantly, in a meta-analysis by Chen et al., the results indicated a significant association of HIF overexpression with increased mortality risk, in terms of overall and disease-free survival, and proved an association of overexpressed HIF-1α with disease progression and unfavorable prognosis in Asian CRC patients [55]. Equally, VEGF mRNA and protein expression levels correlate with vascularity, tumor progression and poor prognosis in CRC [43,50].

Two independent studies on microarray mRNA profiling have revealed that, compared to conventional colon carcinoma (CC), SAC displays a higher representation of hypoxia-related functions and the VEGF pathway and an overexpression and stabilization of HIF-1α [12,24]. HIF-1α stands as one of the most discriminant immunohistochemical biomarkers between SAC and CC, with a positivity of 62.2% and 21.7%, respectively.

Unsurprisingly, in a study aiming to evaluate the functions enriched when comparing differentially methylated genes in SAC versus CC, differentially methylated activities related to VEGF signalling were found, amongst others, to be typically associated with SAC [20].

In the first and only study assessing hypoxia and angiogenesis markers in SAC, Tuomisto et al. revealed that HIF-1α and VEGF expressions and high microvascular density (MVD) were more frequent in SACs than in CCs, thus confirming prior gene expression profiling studies [25]. Specifically, immunohistochemistry analysis of HIF-1α and VEGF in colorectal polyps and colorectal cancers showed that both HIF-1α and VEGF were expressed in most (78–93%) of serrated precursors. In serrated adenocarcinoma, HIF-1α protein was also present in 77.8% of cases, while only 20.3% of CCs were HIF-1α–proficient. VEGF expression significantly correlated with HIF-1α expression in SACs and showed a trend towards a positive association in CCs.

**Figure 3:**
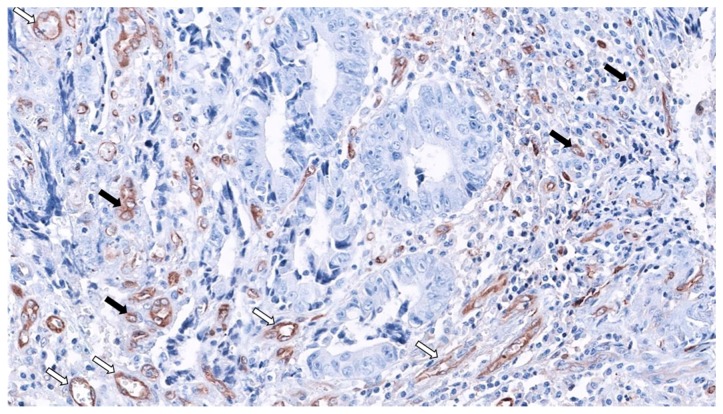
Fascin1 expression in SAC-staining tumor-budding cells (black arrows) and abundant surrounding microvessels (white arrows) 20× original magnification. (Source: Authors).

MVD was significantly higher in SACs, and, surprisingly, serrated morphology was the only significant predictor of MVD in CRC after multivariate analysis. Finally, regarding the mechanism of HIF-1α stabilization, HIF-1α expression was characteristic of well-vascularized tumor in SACs, suggesting a hypoxia-independent mechanism. Besides, in this study, neither BRAF nor KRAS mutation status was associated with HIF-1α and VEGF expressions, nor with HIF-1α stabilization in CC or SACs. Even more, the great majority of SACs carrying wild-type alleles for KRAS and BRAF (81%) were HIF-1α proficient, pointing out to other mechanisms for HIF-1α stabilization in SACs [25]. Figure 3 shows the abundance of SAC-associated MVD using fascin1 immunohistochemistry.

### Therapeutic Opportunities Against Angiogenesis in SAC

Anti-angiogenic therapies aim to improve chemotherapy and radiotherapy treatments by combining with the latter. This improvement is probably due to a maturation effect on blood vessels, a process known as “vascular normalization” [44].

Recently, a number of antineoplastic therapies, such as blocking antibodies of VEGF, inhibitors of low–molecular weight VEGFR, and soluble VEGF constructs (VEGF-Trap), have been developed to neutralize VEGF [56,57]. Indeed, antiangiogenic (anti-VEGF) therapy for mCRC has become the standard therapy [43].

Bevacizumab, a humanized monoclonal antibody binding and neutralizing all human VEGF-A, was the first antiangiogenic drug approved by the US FDA for mCRC treatment in 2004, in combination with chemotherapy in both the first- and second-line setting. Another approach is targeting the receptor instead of the soluble growth factor. In this line, aflibercept is a recombinant fusion protein acting as a soluble decoy receptor binding with high-affinity VEGF-A, VEGF-B, and PIGF, thus inhibiting downstream signalling, and it has been registered as a second-line treatment in combination with FOLFIRI (irinotecan, fluorouracil and leucovorin). Likewise, ramucirumab, a fully humanized monoclonal antibody, selectively binds to VEGFR-2, the main VEGF family receptor involved in angiogenesis, and it received FDA approval in 2015 for the second-line treatment of mCRC (reviewed in [31]).

Regorafenib is a novel oral multikinase inhibitor against the activity of several protein kinases, such as VEGFR-1, VEGFR-2, VEGFR-3, the angiopoietin receptor Tie-2, as well as cKIT, RET, BRAF PDGFR, and FGFR [31]. It was approved by the FDA in 2012 for the treatment of mCRC progressing after failure to available standard treatments. VXM01 is an oral anti-angiogenic vaccine applied as live attenuated *Salmonella* bacteria containing an expression plasmid encoding VEGFR-2, which is currently being tested in a phase I clinical trial in mCRC patients [58]. In a preclinical setting, it has also been described that miR-497 can inhibit CRC metastasis in vitro and in vivo by targeting the VEGF-A/ERK/MMP-9 signalling pathway [59].

At present, other novel anti-angiogenic drugs with various mechanism of action distinct from VEGF(R) inhibition are under clinical investigation, being the first results expected soon [60]. Angiopoietins may also be a valid target for anti-angiogenic drugs. AMG-386 (trebananib) is a peptide-Fc fusion protein that blocks angiogenesis by interfering Ang1 and Ang2 binding to Tie2 receptor. In preclinical studies, trebananib showed anti-tumor activity against colorectal tumor xenografts in mouse [61].

In another line of thought, the introduction of HIF-1α inhibitors in the treatment of CRC patients may be very useful clinically. Cinobufagin suppresses tumor neovascularization by altering the endothelial mTOR/HIF-1α pathway to trigger vascular endothelial cell apoptosis mediated by ROS, and it is emerging as a promising natural anti angiogenic agent [62]. Moreover, there is evidence that the antitumoral effects of the EGFR-blocking antibody cetuximab may be mediated through inhibition of the PI3K pathway, which in turn leads to downregulation of HIF-1α synthesis and activity [50]. Another study suggested that, by targeting the C-terminus of HSP90, it is possible to exploit the prolyl hydroxylase and proteasome pathway to induce HIF-1α degradation in hypoxic tumors [63].

Although there is much evidence reported in this field, resistance to antiangiogenic therapy is still a problem to solve, and many patients do not benefit from anti-angiogenic therapies or develop resistance in the course of treatment, for instance, through the activation and/or upregulation of different pro-angiogenic signals (such as FGF, PDGF, and Ang-1) by anti-angiogenic inhibitors [31]. Predictive biomarkers are needed to identify which patients will develop resistance mechanisms during treatment.

Despite this anti-angiogenic armamentarium and the consistent use of anti-VEGF in metastatic CRC, no studies so far have specifically analyzed whether SAC responds better or worse to anti-angiogenic therapies. Future studies are necessary with the aim of unveiling whether serrated histology could be a predictive marker of anti-angiogenesis response. Nonetheless, despite this lack of knowledge, molecular insights on SAC could give some clues. The most typical molecular alterations associated with the serrated neoplasia pathway could be the mutation in BRAF proto-oncogene [64] and the high level of CpG island methylation phenotype (CIMP-H) [65].

Cytotoxic and anti-angiogenic monoclonal antibody combinations have been tested in *BRAF*^V600E^-mutated patients. FOLFOXIRI (folinic acid, fluorouracil and irinotecan plus folinic acid, fluorouracil, and oxaliplatin) plus bevacizumab is currently considered a potential first-line treatment option for patients with *BRAF*^V600E^–mutated metastatic CRC, given the limitations of standard cytotoxic combinations [32]. Moreover, regorafenib, due to its broad-spectrum kinase inhibitory property, offers benefits in survival in all patient subgroups, including those carrying major oncogene mutations (e.g., RAS and BRAF), as shown in clinical trials [33]. Nowadays, these two molecular therapies in combination with chemotherapy might be the best option for SAC treatment targeting angiogenesis.

On the other hand, CIMP-H arises as a potential predictive biomarker of antiangiogenic treatment efficacy [66]. In 2015, an international consortium developed the Consensus Molecular Subtypes (CMS), classifying CRC into four distinct subgroups [67]. Among these, CMS1 (microsatellite instability immune) tumors are associated with poorer prognosis, high tumor mutational load, MSI, CIMP, BRAF mutation, female gender and right-sided location [67]. Notably, Lenz et al. recently reported that the CMS1 molecular subtype might be a predictive biomarker of response to bevacizumab [68]. This group determined the predictive and prognostic value of the CMS classification of CRC in patients previously enrolled in CALGB/SWOG 80405, a phase III trial that compared the addition of bevacizumab or cetuximab to fluorouracil, leucovorin and oxaliplatin or fluorouracil, leucovorin and irinotecan as the first-line treatment of advanced CRC in a heterogeneous cohort of patients [69]. Analyzing these patients’ outcomes according to their tumor CMS profiles, CMS1 treated with bevacizumab showed better results than treatment with cetuximab. Contrastingly, opposite results were observed for CMS2 (canonical) tumors. These evidences suggest that the CMS classification can be not only prognostic but also predictive of anti-angiogenic treatment efficacy, though further investigations are needed to standardize CIMP status’s definition [31].

## 3. SAC is Especially Capable of Avoiding the Immune Response

The immune response in the serrated pathway plays a critical role in its development, growth and possible treatment response [23,70]. SACs are typically characterized by a weak peritumoral lymphocytic infiltration compared to CC and hmMSI-H tumors [18]. The significance of the relationship between a tumor’s cells and its microenvironment has grown in the last few years through several studies [71]. Among the different components, two of them arise as the major drivers, namely the tumor-associated macrophages (TAMs) and the infiltrating T cells (TILs) [72], while others, such as neutrophils, natural killer cells and dendritic cells, play a supportive role [73,74]. Although the main roles of both TAMs and TILs remain elusive, or even contradictory [75], some discoveries have been made that shed some light on this scenario. On the one hand, in most tumors, TAMs display M2 differentiation, which favors tumor progression and metastases [76] and is correlated with poor prognosis [77] over the M1 differentiation, which, in contrast, leads to an inflammatory response. On the other hand, T cells show a plethora of functions, depending on their type. The T cell response is represented by CD8+ cytotoxic T lymphocytes, the CD4+ T-helper lymphocytes and the Treg cells. While the first have an important antitumoral effect through the release of perforin and granzyme B, the other two types vary in their function, from antitumoral activities to the control of excessive immune response [78].

The gastrointestinal tract continuously interacts with pathogens, developing a well-established immune cohort that contributes to the development and maintenance of the tissue [79]. In colorectal cancer (CRC), this status quo has been driven away, and multiple studies have shown the altered role of the tumor-infiltrating cells, like macrophages, lymphocytes and natural killer cells [71]. Apart from the type of cell, other parameters such as density, location and genetic mutations are key to properly predicting prognosis, patient survival and treatment response [80,81]. Furthermore, immune infiltration tends to improve survival in CRC [82,83], whereas other studies suggest that the interaction between the immune system and the tumor is crucial for the development of distal metastases, the main cause of related colorectal cancer deaths [84]. Here, TAMs arise as driving factors for the development of metastasis, paired with the epithelial-mesenchymal transition (EMT), migration and invasion [72]. These M2 polarized TAMs display a molecular signature that favors positive feedback on tumor cells through the release of anti-inflammatory cytokines [85].

Most SACs are found to be microsatellite-stable tumors [2,3] and thus generate few neoantigens [86]. However, over 10% of SACs are classified as MSI. These tumors bear an important neo-antigen load, which correlates with high immune infiltration [87,88]. Dendritic cells capture these neoantigens and present them on their surface, in MHC-II proteins in lymph nodes, where they activate CD4+ T helper cells, which trigger the activation of CD8+ cytotoxic T cells [89]. Due to this fact, MSI tumors have higher densities of Th1 cells and higher IFN-γ levels than MSS tumors. With this in mind, the so-called immunoscore has been developed in order to categorize the immune infiltration [90]. This is made by quantifying the presence of CD3+ and CD8+ lymphocyte populations in the tumor. This score can have a predictive role in prognosis, e.g., MSI tumors do not benefit from 5-fluorouracyl treatment, display a higher immunoscore and usually have a better prognosis, while some MSSs with good prognosis also have a high immunoscore [91]. However, it is still to be unveiled to what extent microsatellite-unstable SACs are clinically similar to those CRCs without serrated features that show both histological and molecular features of MSI-H, as the diagnostic score for identifying the latter (MSpath score) does not seem valid for the former [92]. These findings show that there is still no good method to predict an accurate prognosis, at least in SAC. Regarding this concern, microarray and methylome analysis comparing CC and SAC has revealed that there are differences between them in terms of immune response. SACs show functional enrichment in phagocytosis, B-cell response and a down-representation of IL-12 pathways [12]. The increase of the B cell response can lead to an inhibition of the anti-tumor immunity [93], while the inhibition of IL-12 shifts the immune environment towards anti-inflammatory behavior [94]. This is also demonstrated by the fact that HIF-1α, whose expression is higher in SAC than in CC [24,25], has been associated with the development of an immunosuppressive microenvironment in the tumors [95]. The actin-bundling protein fascin1, which is also overexpressed in SAC [92], could be implicated as well in the regulation of the immune response, via suppressing RIG-I signalling and IFN-β production, and therefore preventing immune-related cell death [96]. In addition to this, the study of methylation of SAC compared to hm-MSI-H has shown differences in methylation and in the expression of some immune-related genes, such as CD14, TLR4 and HLA-DOA. CD14 and HLA-DOA tend to be more methylated in hmMSI-H than in SAC. These genes, along with CXCL14, are upregulated in SAC compared to hmMSI-H, although the cell of origin for this overexpression is elusive, as these genes are normally expressed in myeloid cells, which are not as abundant in SAC as they are in CC or hmMSI-H [27]. ICAM1, however, is more expressed in hmMSI-H than in SAC, which typically favors antigen presentation, is associated with less risk of metastases and is related with a good prognosis due to its association with leukocyte extravasation and improved immune response in the tumor microenvironment [6]. The study of the microtranscriptome has shown a difference in the expression of miR-181-a2, which is lower in SAC than in hmMSI-H. The lower levels of this mircroRNA regulate the immune response by enhancing the expression of IL-2, IL-22 and IL-17a in the serrated lesions, which then might favor an immunosuppressive phenotype [28,97]. These differences confirm the importance of understanding the immune environment in SAC. Along these lines, a new classification of SAC has recently been proposed. The “classical” serrated CRC shows MSI-H and strong immune infiltration, thus making this subtype a good candidate for immuno-checkpoint inhibition therapy (ICI). In contrast, the DKOIEC mouse model, which recapitulates the human “mesenchymal” serrated, displays immunosuppressive behavior, high levels of PD-L1 expression and exclusion of CD8+ cells from the tumor [22]. The future understanding of serrated lesions and their microenvironment would provide better tools for diagnosis and treatment.

### Therapeutic Approaches for Avoiding Immune Evasion in SAC

In the last few years, the rise of immunotherapies based on the modulation of the immune response has opened a new horizon in tumor treatment therapeutics. These therapies’ objective is to enhance T cell activation, targeting three main surface proteins: CTLA4, PD-1 and PDL-1. Despite the fact that immunotherapies against CRC have not rendered results as good as in other cancers like melanoma, considerable progress has been made in that field. As commented previously, the majority of SACs are MSS, this feature normally implying a low response to immune checkpoint treatments. Thus, in theory, only MSI-H “classical” SACs would be suitable for antibody therapies at the moment. CTLA4 (Cytotoxic T-lymphocyte antigen 4) is a membrane glycoprotein that inhibits T cell response and is key to the phenomenon of immune tolerance. Its blockade with monoclonal antibodies such as ipilimumab and tremelimumab induces T cell proliferation and increases IL-2 production and depletion of T regulatory lymphocytes in the tumor microenvironment [34].

PD-1 (Programmed Death-1 receptor) or CD279 is an inhibitory co-receptor that is expressed on the surface of CD8+ CTLs, NK cells and tumor-infiltrating lymphocytes [35]. Nivolumab and pembrolizumab are currently the available monoclonal antibodies approved by the FDA. The PD-1 blockade restores the CD8+ lymphocytes’ infiltration and triggers the reduction of the tumor’s size. In the case of pembrolizumab, the efficacy of these treatments has led to their rapid approval for patients with no alternative treatment options. Some studies have shown the high expression of PD-1 in MSI-H tumors, thus providing the evidence for including its immunohistochemical expression as a predictive marker [36,37].

PD-L1 (Programmed Death Ligand-1 receptor) or CD274 is expressed on the surface of certain activated immune components, such as B cells, T cells and NK cells CD14+-TAMs, and on the surface of cancer cells [98,99]. The interaction between PD-1 and PD-L1 leads to the inhibition of T cell activation and reduces pro-inflammatory cytokine secretion (INF-γ, TNF-α and IL-2). Normally, this expression is necessary to avoid autoimmunity, but in certain types of cancer, it results in tumor growth and lack of adaptive immune response due to the fact that CTLs cannot properly attack cancer cells. At the moment, the available monoclonal antibodies are MDX1105, durvalumab, avelumab and atezolizumab. MDX 1105 is still in phase I study, while the rest have been approved by the FDA [35]. MDX1105 has been demonstrated to be unsuitable for CRC treatment, while the response rate of atezolizumab in CRC is still low. Despite the failure of monotherapies, preclinical and phase I clinical studies consisting of the co-administration of anti-PD-L1 with inhibitors of the TGFβ receptor (galunisertib) or MEK inhibitors and atezolizumab offer promising therapeutic approaches [22].

## 4. SAC Displays an Active Invasive Front

Compared to CC, SAC exhibits a higher occurrence of adverse histological and molecular features at the invasive front, including high-grade tumor budding (HG-TB), cytoplasmic pseudofragments and an infiltrating growth pattern [18]. The invasive front comprises a dynamic process of reprograming of colorectal carcinoma cells known as epithelial mesenchymal transition (EMT) [100]. EMT is a process in which epithelial cells lose cell polarity and cell–cell adhesion favored by downregulation of the epithelial cell adhesion molecule E-cadherin. EMT is triggered by several transcription factors, such as SNAIL and SLUG which induced cells to acquire migratory and invasive properties. In normal cells, EMT contributes to several physiological processes, such as wound healing and embryogenesis [101]. However, in cancer cells, EMT is associated with the development of tumor phenotypes, including invasion, metastasis and drug resistance [102,103]. EMT can be identified histologically by the presence of tumor budding (TB), a manifestation that is specific to tumors showing an infiltrating growth pattern [104]. The reported incidence of TB in CRC varies widely in the literature from 20% to 89%, being this wide range possibly as a result of different diagnostic criteria and quantification methods [20]. In this line, García-Solano et al. described that SACs had more high-grade TB than CCs (69.1–40.7%, *p* = 0.0003) [18], this phenomenon being associated with lower E-cadherin expression and higher of mesenchymal markers [19]. It is known that the implication of the Wnt signalling pathway in the main process of TB formation is usually induced by increased expression of nuclear β-catenin [105]. Apart from being a transcription factor, β-catenin is a structural adaptor that links cadherins to cytoskeletal actin, thus participating in cell–cell adhesion [106]. Nuclear β-catenin expression was absent in 78.4% of SACs, and this percentage was significantly higher than that observed in CCs (39.6%) (*p* < 0.0001), thus suggesting a lack of involvement of this mechanism in the EMT in SAC [20].

Given the heterogeneity of CRC in terms of clinical behavior, great efforts have been made to identify histological features that may help forecast the aggressiveness of a given CRC and hence select patients for closer monitoring and/or more aggressive treatment [104]. Cytoskeleton rearrangements are necessary for tumor cells to acquire an invasive phenotype. Studies on the role of actin and its interacting partners have underlined key signalling pathways, such as the Rho GTPases, and effector proteins that, through the cytoskeleton, facilitate tumor cell migration, invasion and metastasis [107]. The three most widely studied Rho GTPases in eukaryotic cells, Rho, Rac and Cdc42, control the assembly of the actin cytoskeleton, and Cdc42 that of the microtubule cytoskeleton [108]. In general terms, Rho can recruit the ROCK (Rho-associated coiled-coil forming protein kinase, or Rho kinase) family of kinases [109], which regulates various cytoskeletal proteins, inducing actin stress fibre formation and the generation of contractile forces [108]; Rac rearranges the actin cytoskeleton to promote formation of membrane protrusions, called lamellipodia, which drive motility in different cell types; and Cdc42 signalling favors the generation of actin-rich microspikes to sense extracellular chemotactic gradients and trigger directed cell movement. A main downstream effector of the Rho GTPase family is Rho kinase, which plays a crucial role in the regulation of actin remodelling via phosphorylation of cofilin and myosin light chain (MLC).

Evidences on differentially enriched functions and genes seem to demonstrate that SAC, unlike CC, has a characteristic profile of cytoskeletal rearrangement, which could account for particular cell adhesion and invasive properties. In fact, specific activation of pathways related to small GTPases and second messengers such as phosphatidylinositols are frequently found as enriched functions in SAC [12,20]. In this line, the roles of phosphatidylinositol trisphosphate and the small GTPases RAC and CDC42 in actin assembly are important for lamellipodia and filopodia rearrangement [110].

Fascin1 emerged as an immunohistochemical marker for SAC diagnosis, as the positive expression of fascin1 was observed in 88.6% of SACs and in 14.3% of CCs (88.6% sensitivity, 85.7% specificity) [12]. Intriguingly, Tao et al. had previously observed that β-catenin is associated with the actin-bundling protein fascin in a non-cadherin complex. In fact, these authors observed that fascin1 and E-cadherin use a similar binding site within beta-catenin and that fascin and beta-catenin co-localize at cell–cell boundaries and dynamic cell-leading edges of epithelial and endothelial cells [111], thus giving an explanation of why β-catenin was not observed in the nuclear location in SACs. Fascin1 protein localizes to the core actin bundles forming spikes and filopodia at the leading edge of migratory cells, increasing migration in several cell types [112] and therefore, it has been associated with adverse prognosis in CRC [113]. These facts can partly explain the higher incidence of adverse prognostic histologic factors at the invasive front of SAC (HG-TB and weak peritumoral lymphocytic infiltration (PLI)) and the worse outcomes observed in SACs [18]. Additionally, fascin1 expression is associated with shorter survival, as has been reported previously in CRC [114], thus supporting earlier observations showing that SAC fares worse than CC [113]. Induced expression of fascin1 in colorectal cancer cells increased migration and invasion in cell cultures and caused cell dissemination and metastasis [115]. Figure 3 shows the expression of fascin1 in both blood vessels and SAC cells at the invasive front, creating tumor budding.

### Possible Therapies Targeting SAC Invasive Front

Migrastatin analogues, such as macroketone, have been shown to inhibit metastatic tumor cell migration, invasion via fascin1 blockade [38], thereby suggesting a possible role for migrastatin analogues in SAC treatment [12]. However, the complex structure of the macroketone hinders its synthesis, and other anti-fascin1 compounds derived from indazol-furan-carboxamides have been tested [38]. Huang et al. showed that G2 compound inhibits the actin-bundling function of fascin1 and blocks tumor cell migration, invasion and metastasis in breast tumor cells [116]. In this line, Montoro and Alburquerque et al. prove the in vitro and in vivo anti-tumoral activity of G2 compound on colorectal cancer cells and guide to design improved G2-based fascin1 inhibitors [39]. On the search for new anti-fascin1 drugs, this same group performed an in silico screening of 9591 compounds, including 2037 approved by the FDA, for the purpose of analyzing their fascin1 binding affinity. The screening results yielded the FDA-approved antidepressant imipramine as the most evident potential fascin1 blocker. This tricyclic antidepressant (TCA) has an anti-invasive and anti-metastatic activity in a dose-dependent manner more evident in fascin1-overexpressing colorectal cell lines, in both constitutive and induced fascin1 expression [40]. A previous study by Jahchan et al. also demonstrated the antitumoral effects of imipramine in human small-cell lung cancer and other neuroendocrine tumors implanted in mice [117]. In a clinical setting, Sauer and Jansen reported unexpected survival associated with imipramine treatment in a patient with metastatic lung cancer [41], and the epidemiological study by Walker et al., which included 31,953 cancer cases from different locations and 61,591 matched controls, concluded that tricyclic antidepressants like imipramine may have the potential to prevent both colorectal cancer and glioma in a dose- and time-dependent fashion [42]. All this evidence paves the way for a potential molecular targeted therapy for SAC and other fascin1-overexpressing tumors.

## 5. Concluding Remarks

This molecular classification of CRC provides a better understanding of the insights on the biology of different tumor subtypes, with the aim of forecasting the clinical behaviour and possible therapeutic targets for a given tumor. However, this approach is far from the routine practice, as most CRCs are diagnosed based on histological grounds. With this in mind, the association of molecular features with each histological subtype is crucial for the proper clinical management of CRC patients. SAC is defined by a series of morphological criteria and associated with histologically adverse factors present at the tumor’s invasive front. At the molecular level, the activation of the MAPK pathway (either by KRAS or BRAF mutation), the prominent EMT (not involving nuclear β-catenin expression) and the frequent CIMP-H status are characteristic of the tumor itself, whereas the abundance of microvascular density and the weak immune response are common features of the surrounding tumor microenvironment. All these findings give us clues about possible ways to treat this aggressive tumor, yet future studies, including preclinical trials using available serrated mouse models, clinical trials testing FDA-approved drugs against SAC targets or simply retrospective analysis based on histological features associated to the response to specific treatments, are needed.

## Figures and Tables

**Table 1:** Potential therapeutic approaches to treat tumors with SAC histology or SAC-associated biomarkers.

	Potential Treatment	Target(s)	FDA Approved	Treatment Current Indication	Rationale for Indication in Serrated Cancers	Reference(s)
Anti-angiogenic therapy	Bevacizumab + chemotherapy	VEGF-A	Yes	mCRC	SAC presents higher expression HIF-1, VEGF and MVD compared to CC	[31]
	Ramucirumab + FOLFIRI	VEGFR-2	Yes	mCRC		[31]
	Aflibercept in combination with FOLFIRI	VEGF-A, VEGF-B and PIGF	Yes	mCRC		[31]
	Bevacizumab + FOLFOXIRI	VEGF-A	Yes	mCRC*		[32]
	Regorafenib	VEGFR-1, VEGFR-2, VEGFR-3, Tie-2,cKIT, RET, BRAF PDGFR, and FGFR	Yes	mCRC		[31,33]
Therapy for avoiding immune response	Anti-PD-L1 and Galunisertib	PD-L1 and TGFβR	Yes	MSS-SAC	High PD-L1 expression, lack of CD-8+ response	[22]
	Atezolizumab and MEK inhibitors	PD-L1 and MEK	Yes	MSS-SAC		[22]
	Ipilimumab and tremelimumab	CTLA-4	Yes	MSIH classical SACs	Possible utility in classical SAC based on MSI-H status	[34]
	MDX1105, durvalumab, avelumab and atezolizumab	PD-L1(CD274)	Yes, MDX1105 in phase I study	MSIH classical SACs		[35]
	Nivolumab and pembrolizumab	PD-1(CD279)	Yes	MSIH classical SACs		[36,37]
Possible therapies targeting SAC invasive front	Migrastatin	Fascin1	No	None	Fascin1 overexpression in SAC Synthetic drug FDA approved drug	[38]
	G2	Fascin1	No	None		[39]
	Imipramine	Fascin1and GPCRs (G protein coupled receptors)	Yes	Antidepressant		[40,41,42]

CTLA-4: cytotoxic T lymphocyte-associated Ag-4, MEK: MAPK/extracellular signal-regulated kinase, mCRC; metastatic colorectal cancer, SAC: serrated adenocarcinoma, MSI-H: high level of microsatellite instability, MSS: microsatellite stable, MVD: microvascular density. FGFR: fibroblast growth factor receptor, FOLFIRI: irinotecan, fluorouracil and leucovorin. FOLFOXIRI: folinic acid, fluorouracil and irinotecan and oxaliplatin. GPCRs: G protein coupled receptors, PDGFR: platelet-derived growth factor receptor, PlGF: placental growth factor, TGFβR: transforming growth factor receptor β, VEGF: vascular endothelial growth factor type A, VEGFR: VEGF receptor. *Reasonable option for mCRC *BRAF*^V600E^ mutated patients.

## Supplementary Materials

Supplementary materials can be found at https://www.mdpi.com/1422-0067/21/6/1991/s1. Figure S1. Plot showing the 10 significant pathways with the most affected genes after the enrichment analysis of the 2924 genes shared by CC and SAC. The pathways in cancer are the most represented, with 79 affected genes. In this chart, we can observe that these 79 genes are also affected by other pathways related to other types of cancer, the cell cycle and some metabolic processes. Other interesting pathways were also over-represented, such as RNA degradation and the p53 signalling pathway.
