# Supplementary material for: Biology and Therapeutic Targets of Colorectal Serrated Adenocarcinoma; Clues for a Histologically Based Treatment against an Aggressive Tumor
Source: Int J Mol Sci. 2020 Mar 14;21(6):1991. doi: 10.3390/ijms21061991 (PMC7139914; doi:10.3390/ijms21061991)
Supplement: Supplementary file 1 [file ijms-21-01991-s001.pdf]

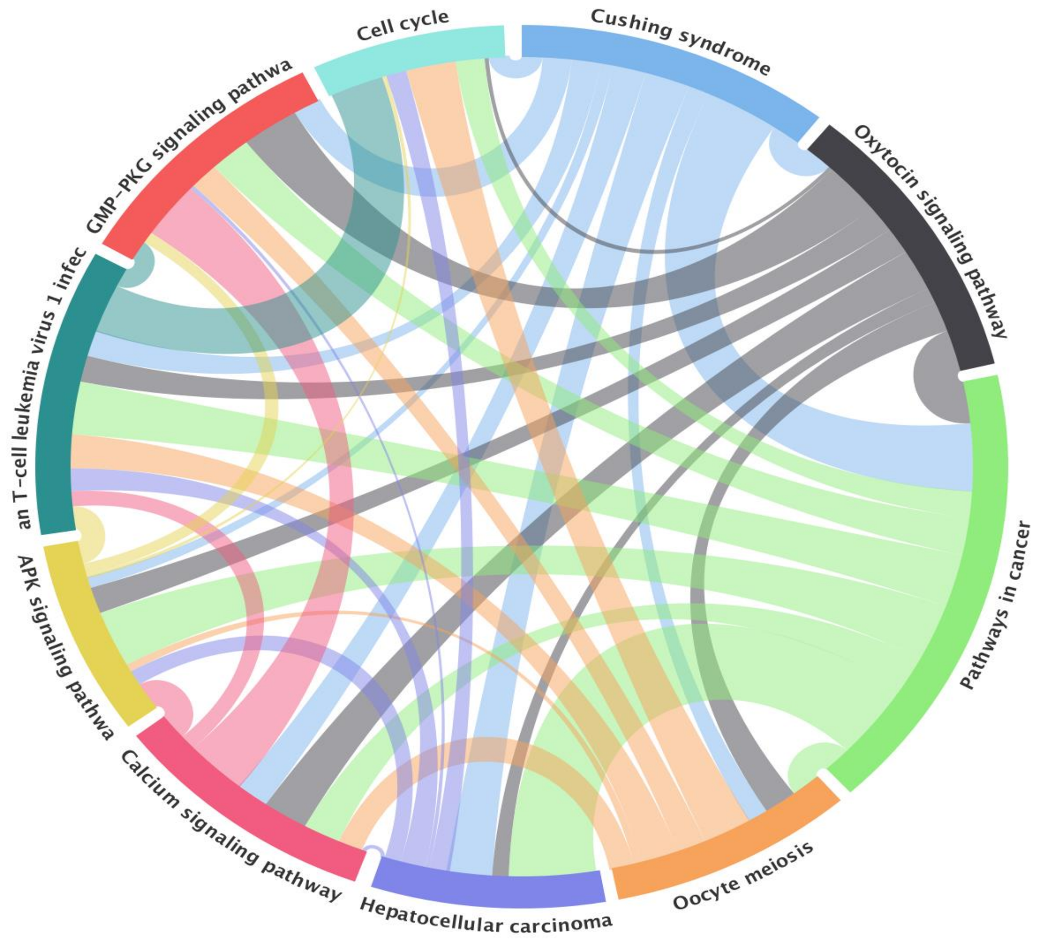

Figure S1. Plot showing the 10 significant pathways with the most affected genes after the enrichment analysis of the 2924 genes shared by CC and SAC. The pathways in cancer are the most represented, with 79 affected genes. In this chart, we can observe that these 79 genes are also affected by other pathways related to other types of cancer, the cell cycle and some metabolic processes. Other interesting pathways were also over-represented, such as RNA degradation and the p53 signalling pathway.
